# Efficacy and Safety of Massage Therapy for Cognitive Impairment in Patients With Autism Spectrum Disorder: Protocol for a Systematic Review and Meta-Analysis

**DOI:** 10.2196/88284

**Published:** 2026-04-13

**Authors:** Yi Zhong, Sijie Dang, Xiaoqiu Wang, Xin Yun Chia, Jing Zhang, Weijiang Li, Chongjie Yao, Zhengyu Li, Bin Xiao

**Affiliations:** 1 School of Acupuncture-Moxibustion and Tuina, Shanghai University of Traditional Chinese Medicine, No. 1200 Cailun Road, Shanghai, 201203, China, 86 17321311968

**Keywords:** autism spectrum disorder, massage therapy, cognitive impairment, systematic review, meta-analysis, protocol

## Abstract

**Background:**

Autism spectrum disorder (ASD) is a neurodevelopmental condition marked by social communication deficits and cognitive impairment. As pharmacological and behavioral treatments often show inconsistent efficacy, massage therapy—a low-risk complementary approach including *tuina* and acupressure—is used to improve cognitive symptoms, yet its systematic efficacy and safety remain unevaluated.

**Objective:**

This systematic review and meta-analysis aims to evaluate the efficacy and safety of massage therapy (categorized into traditional Chinese medicine–based and Western-based approaches) for improving specific cognitive domains (eg, executive function, attention, and memory) in individuals with ASD.

**Methods:**

Randomized controlled trials (RCTs) and cluster RCTs will be identified across 9 English and Chinese databases (including PubMed, Cochrane Library, and China National Knowledge Infrastructure) through September 2025. Two reviewers will independently perform study selection, data extraction, and risk-of-bias assessment using version 2 of the Cochrane risk-of-bias tool. A random-effects model will be primarily applied due to anticipated clinical diversity, with fixed-effects models reserved for sensitivity analyses of homogeneous data (*I*^2^=0%). Subgroup analyses will explore age, intervention context, and massage type. Evidence for primary outcomes—including checklist scores and specialized cognitive scales—will be assessed using the Grading of Recommendations Assessment, Development and Evaluation (GRADE) approach.

**Results:**

Selection results will be summarized in a PRISMA (Preferred Reporting Items for Systematic Reviews and Meta-Analyses) flow diagram. This review will synthesize evidence on massage therapy’s effects on cognitive outcomes measured using scales such as the Autism Behavior Checklist, Childhood Autism Rating Scale, and Autism Treatment Evaluation Checklist, as well as safety profiles. This study was funded in 2024. Following the September 2025 search, analysis will be completed by June 2026, with final results expected for submission in December 2026.

**Conclusions:**

This study will evaluate massage therapy’s role in managing cognitive impairment in ASD. By synthesizing available evidence from RCTs, the findings will provide an evidence-based foundation for clinical decision-making and clarify whether manual therapies serve as safe, effective nonpharmacological adjuncts to existing ASD interventions.

## Introduction

### Background and Significance

Autism spectrum disorder (ASD), commonly known as autism, is a neurodevelopmental disorder characterized by early onset during childhood. Its core clinical features include impairments in social communication, repetitive stereotyped behaviors, and restricted interests [1]. According to the latest 2025 data from the China Disabled Persons’ Federation, the total number of individuals with ASD in China ranges from 13 to 14 million, among whom 3 to 5 million are children aged 0 to 14 years, accounting for one-fifth of the global pediatric ASD population. As stated in the 2022 annual report of the Autism and Developmental Disabilities Monitoring Network released by the US Centers for Disease Control and Prevention on April 15, 2025, the prevalence of ASD among children in the United States reached 3.23% (1 in 31) in 2022, representing a 15% increase compared to 2020 (1 in 36) [2].

ASD typically manifests in early childhood and is associated with significant cognitive dysfunction, behavioral disturbances, and language developmental delays [3]. This situation not only severely impacts patients’ normal social interactions and physical and mental well-being but also imposes a substantial caregiving burden and economic strain on families and society [4-9].

The etiological mechanisms of ASDs remain incompletely understood and may be associated with genetic factors and environmental risks [10-12]. Clinically, treatment primarily relies on symptom-targeted individualized regimens, and there are still no specific curative interventions. Current rehabilitative interventions are mainly categorized into pharmacological and nonpharmacological approaches [13,14]. While Western medication and behavioral training are widely used, they are limited by inconsistent efficacy and significant adverse effects [15-19]. Notably, an increasing number of clinical studies have focused on traditional Chinese medicine (TCM) for ASD treatment in recent years, offering a novel direction for ASD rehabilitation [20-23]. Compared to other complementary therapies in TCM, massage therapy has greater acceptance [24].

Recent clinical and mechanistic studies provide compelling evidence on massage therapy in ASD. First, the *tuina* intervention can significantly reduce core symptom scores (Autism Behavior Checklist [ABC] and Childhood Autism Rating Scale [CARS]) and improve overall clinical efficacy [25]. The therapeutic mechanisms appear multifaceted, involving the modulation of key neuroplasticity pathways such as the PI3K/AKT and BDNF-CREB in animal models [26,27], as well as the activation of social brain regions and oxytocin release in humans [28]. Furthermore, manual techniques may enhance the central nervous system environment by promoting fluid clearance [29]. A systematic review found that massage can improve self-regulation difficulties and emotional distress in pediatric patients. Through rhythmic tactile stimulation, massage may exert autonomic nervous system regulation and psychological relaxation effects on children, thereby contributing to symptom relief to a certain extent [30].

Although a considerable number of clinical studies have explored massage therapy for ASD, there is a lack of systematic reviews or meta-analyses synthesizing evidence on its efficacy and safety, hindering the establishment of reliable evidence-based foundations. Previous meta-analyses on massage therapy for ASD have emphasized behavioral symptoms and stress [30]. However, cognitive impairments—which are a major cause of disability in ASD—remain underinvestigated in the context of manual therapies. This study is novel in its exclusive focus on cognitive impairment as the primary outcome, aiming to provide the first systematic synthesis of evidence regarding the effects of massage therapy on specific cognitive functions and systematically consolidating evidence to address the research gap in nonpharmacological cognitive interventions for ASD. Therefore, we aim to address this issue through this systematic review and meta-analysis.

### Research Objectives

This study protocol describes a systematic review and meta-analysis of published randomized controlled trials (RCTs) investigating massage interventions for ASD, aiming to objectively evaluate the clinical efficacy of massage therapy in improving cognitive impairment in ASD through data synthesis and collation. This review protocol is registered in the PROSPERO platform with the registration number CRD420251038194 [31].

## Methods

This protocol follows the PRISMA-P (Preferred Reporting Items for Systematic Reviews and Meta-Analyses Protocols) guidelines [31].

### Eligibility Criteria (Population, Intervention, Comparator, Outcome, and Study Design Framework)

#### Participants

The review will focus on patients of any age diagnosed with ASD using standardized criteria (eg, the *Diagnostic and Statistical Manual of Mental Disorders, Fifth Edition*; *International Classification of Diseases, 10th Revision*; or their latest revisions, the *Diagnostic and Statistical Manual of Mental Disorders, Fifth Edition, Text Revision* and *International Classification of Diseases, 11th Revision*). Given that the critical window for cognitive development in ASD is childhood and intervention responses vary significantly across developmental stages (eg, infants vs adolescents vs adults), age will be treated as a primary factor in our analytical framework.

To minimize confounding effects on cognitive assessment, studies involving participants with specific conditions will be excluded. These exclusions comprise individuals with severe neurological comorbidities, such as active epilepsy defined as 2 or more seizures in the previous 3 months, cerebral palsy, or known structural brain abnormalities. We will also exclude participants with severe intellectual disability, characterized by an IQ below 50 as measured using standardized scales such as the Wechsler Intelligence Scales.

Furthermore, individuals with major psychiatric disorders, including schizophrenia or bipolar disorder, and those with significant sensory impairments, such as severe visual or hearing loss that would preclude valid cognitive testing, will be ineligible.

Finally, the review will exclude patients with severe physical illnesses or unstable health conditions, such as uncontrolled cardiovascular or pulmonary disease, liver or kidney failure, malignant tumors, or the active phase of severe autoimmune diseases.

Textbox 1 summarizes the specific inclusion and exclusion criteria for participants designed to ensure a focused assessment of massage therapy’s effects on cognitive function.

Textbox 1. **Summary of population, intervention, comparator, outcome, and study design–based inclusion and exclusion criteria for evaluating the effects of massage therapy on cognitive function in individuals with autism spectrum disorder (ASD).**

**Inclusion criteria**

- Patients of any age diagnosed with ASD using standardized criteria (eg, *Diagnostic and Statistical Manual of Mental Disorders, Fifth Edition* (*DSM-5*); *International Classification of Diseases, 10th Revision*; or their latest revisions, *DSM-5 Text Revision* and *International Classification of Diseases, 11th Revision*)
- Focus on developmental stages (childhood, adolescence, or adulthood) as a primary analytical factor

**Exclusion criteria**

- Severe neurological comorbidity: active epilepsy (≥2 seizures in the previous 3 months), cerebral palsy, or known structural brain abnormalities
- Severe intellectual disability: defined as an IQ below 50 measured using standardized scales (eg, Wechsler Intelligence Scales)
- Major psychiatric disorders: such as schizophrenia or bipolar disorder
- Significant sensory impairments: severe visual or hearing loss that would preclude valid cognitive testing
- Severe physical illness or unstable health conditions: uncontrolled cardiovascular or pulmonary disease, liver or kidney failure, malignant tumors, or active severe autoimmune disease

The presence of these excluded conditions, such as severe neurological comorbidities and intellectual disability, may independently and profoundly impact cognitive function, thereby obscuring the specific intervention effect of *tuina* and massage therapy.

#### Intervention

For the experimental group, massage therapy is defined as any systematic manual manipulation of soft tissues, including *tuina*, acupressure, or Swedish massage, performed by a trained professional for therapeutic purposes to provide a comprehensive evidence map of manual therapies for ASD cognitive symptoms. To ensure consistency and clinical relevance, studies must meet *minimum operational criteria* for *tuina* as recommended by the technical specifications for pediatric *tuina* in medical institutions [32].

Specifically, the intervention must be described as “*tuina*” or “pediatric *tuina*” and incorporate fundamental maneuvers defined in the specifications, such as kneading, pressing, pushing, and rubbing performed by a trained practitioner (eg, *tuina* therapist or physician) on specific body regions (eg, head, back, and limbs) or acupoints (eg, Baihui DU20 and Sishencong EX-HN1).

Furthermore, each treatment session should typically last between 15 and 30 minutes to align with standard clinical practice, and studies must clearly report the treatment frequency and total intervention period to facilitate subgroup analysis of the dose-effect relationship.

Other massage therapies (eg, Swedish massage, Thai massage, shiatsu massage, and acupressure) will also be included provided they offer clear descriptions of the techniques applied. These interventions will be categorized into TCM based (eg, *tuina* and acupressure) and Western based (eg, Swedish and myofascial release) for subsequent analysis. Studies lacking a detailed description of the manual procedures will be categorized as insufficiently described *tuina* for the purpose of subgroup analysis.

Massage therapy could be administered as a stand-alone treatment or as an adjunct to conventional care (eg, behavioral therapy and special education). Cointerventions must be reported and will be accounted for in the analysis.

#### Comparators

The control groups will include any of the following: no treatment, waitlist, placebo or sham interventions (eg, light touch or simulated massage without therapeutic techniques), or usual care or standard of care (eg, routine behavioral therapy and special education).

Studies using other active interventions (eg, pharmacological treatments) as a comparator will also be included provided the experimental group receives massage therapy as an adjunct to the same standard care.

Studies in which massage therapy is administered to the control group will be excluded.

#### Outcomes

##### Primary Outcomes

The primary outcomes of this study involve core ASD symptom severity and cognitive impairment measured using standardized scales, with a focus on extracting domain or item scores that most directly reflect cognitive and language functions. For the ABC, the language and cognitive domain scores will be extracted. For the CARS, the analysis will extract the score from the specific item assessing cognitive function. For the Autism Treatment Evaluation Checklist (ATEC), the total score of the “cognitive/language” module will be used. Furthermore, if a study reports only the total score of these scales but not the specific domain scores, the total score will be recorded and included in a separate sensitivity analysis to assess its consistency with the domain-specific findings.

##### Secondary Outcomes

Secondary outcomes for this review will include direct assessments of specific cognitive domains using specialized tools to improve the targetedness and accuracy of the evaluation. These assessments will encompass executive function measured using instruments such as the Behavior Rating Inventory of Executive Function; attention measured via standardized continuous performance tests; and memory or general cognitive ability measured through IQ scores, Wechsler Intelligence Scales, or the Leiter International Performance Scale–Revised.

The clinical efficacy rate will also be evaluated, with the decision to perform a meta-analysis on clinical efficacy rates depending primarily on the clinical comparability of the definitions used across the studies. Rather than applying arbitrary numerical cutoffs for efficacy definitions, we will qualitatively evaluate the criteria for response or marked improvement, conducting quantitative synthesis only if these definitions are deemed clinically similar enough to yield a meaningful pooled effect. If substantial clinical variation exists, such as significantly different diagnostic thresholds, we will perform subgroup analyses based on the stringency of the definition or provide a descriptive qualitative synthesis.

Additionally, we will record physiological markers such as serum levels of tissue plasminogen activator and plasminogen activator inhibitor-1 alongside any reported undesirable effects such as discomfort, crying, or skin irritation during or after the massage sessions.

Finally, long-term outcomes, including cognitive scores or quality of life measures assessed at follow-up time points (eg, 1 or 3 months after treatment) will be systematically analyzed.

### Study Design

We will include RCTs, including both parallel-group and cluster RCT designs. For cluster RCTs, we will extract data adjusted for clustering if reported. If not, we will approximate the effective sample size using an intracluster correlation coefficient from similar studies or perform sensitivity analyses using a range of plausible intracluster correlation coefficient values. Nonrandomized studies, case reports, case series, reviews, protocols, and studies with incomplete or unavailable outcome data will be excluded.

### Information Sources and Search Strategy

#### Databases

The comprehensive literature search was conducted from database inception to September 30, 2025, across 5 English-language databases and 4 Chinese-language databases. English-language resources included PubMed, Web of Science, Scopus, Cochrane Library, and Embase, whereas Chinese-language resources encompassed China National Knowledge Infrastructure (CNKI), Wanfang Data, VIP Chinese Scientific Journal Database, and the Chinese Biomedical Database.

To identify ongoing or recently completed trials, we systematically searched clinical trial registries, specifically ClinicalTrials.gov and the Chinese Clinical Trial Registry.

The search identified varying numbers of records across these platforms, including 8 results from PubMed, 37 from Web of Science, 24 from Embase, 161 from the Cochrane Library, 49 from ClinicalTrials.gov, 100 from the Chinese Biomedical Database, 51 from CNKI, 78 from Wanfang Data, and 86 from VIP Chinese Scientific Journal Database. A detailed representative search strategy for PubMed is presented in Table 1 for reference.

**Table 1.** Detailed search strategy for PubMed (executed in September 2025).

Search number	Query	Results, n
1	“Autism Spectrum Disorder”[Mesh]	49,279
2	(((((((((((((((((((((((Autistic Spectrum Disorder[Title/Abstract]) OR (Autistic Spectrum Disorders[Title/Abstract])) OR (Disorder, Autistic Spectrum[Title/Abstract])) OR (Autism Spectrum Disorders[Title/Abstract])) OR (ASD(Autism Spectrum Disorder[Title/Abstract]))) OR (Autistic Disorder[Title/Abstract])) OR (Disorder, Autistic[Title/Abstract])) OR (Disorders, Autistic[Title/Abstract])) OR (Autism[Title/Abstract])) OR (Autism, Early Infantile[Title/Abstract])) OR (Early Infantile Autism[Title/Abstract])) OR (Infantile Autism, Early[Title/Abstract])) OR (Autism, Infantile[Title/Abstract])) OR (Infantile Autism[Title/Abstract])) OR (Kanner Syndrome[Title/Abstract])) OR (Pervasive Developmental Disorder[Title/Abstract])) OR (PPD(Pervasive Developmental Disorder[Title/Abstract]))) OR (Asperger Syndrome[Title/Abstract])) OR (Childhood Disintegrative Disorder[Title/Abstract])) OR (Atypical Autism[Title/Abstract])) OR (Child Development Disorders, Pervasive[Title/Abstract])) OR (Pervasive Child Development Disorders[Title/Abstract])) OR (Pervasive Development Disorders[Title/Abstract])) OR (Autism Spectrum Disorder[Title/Abstract])	76,574
3	“Massage”[Mesh]	7355
4	((((((((((((((((((((((Massage Therapy[Title/Abstract]) OR (Massage Therapies[Title/Abstract])) OR (Therapies, Massage[Title/Abstract])) OR (Therapy, Massage[Title/Abstract])) OR (Myofascial Release Therapy[Title/Abstract])) OR (Myofascial Treatment[Title/Abstract])) OR (Myofascial Release[Title/Abstract])) OR (Myofascial Release Treatment[Title/Abstract])) OR (Acupressure[Title/Abstract])) OR (tuina[Title/Abstract])) OR (Musculoskeletal Manipulations[Title/Abstract])) OR (Craniosacral Massage[Title/Abstract])) OR (Manipulation Therapy[Title/Abstract])) OR (Manipulation Therapies[Title/Abstract])) OR (Manipulative Therapies[Title/Abstract])) OR (Manipulative Therapy[Title/Abstract])) OR (Manual Therapies[Title/Abstract])) OR (Manual Therapy[Title/Abstract])) OR (Chiropractic Manipulation[Title/Abstract])) OR (Chiropractic Spinal Adjustment[Title/Abstract])) OR (Osteopathic Manipulation[Title/Abstract])) OR (Osteopathic Manipulative Treatment[Title/Abstract])) OR (Massage[Title/Abstract])	22,163
5	((Randomized Controlled Trial[Title/Abstract]) OR (Randomized Controlled Trials[Title/Abstract])) OR (RCT[Title/Abstract])	167,976
6	Search 1 OR 2	82,335
7	Search 3 OR 4	24,629
8	Search 5 AND 6 AND 7	8

#### Search Terms

The search terms integrated 3 primary concepts, including ASD-related terms, massage-related interventions such as *tuina* and manual therapy, and cognitive-related outcomes, combined with specific filters for RCTs.

#### Supplementary Search

Furthermore, to minimize publication bias and identify ongoing or recently completed studies, we conducted supplementary searches for gray literature, including conference proceedings from major international and domestic conferences in the fields of autism research and complementary medicine, such as the International Meeting for Autism Research and key Chinese academic conferences on *tuina* or manual therapy. Academic dissertations were searched within the thesis repositories of the aforementioned Chinese and English databases, including CNKI, Wanfang Data, and ProQuest Dissertations and Theses Global. The reference lists of all included studies and relevant systematic reviews were manually scanned for additional eligible studies.

### Study Selection and Data Extraction

#### Study Selection

The study selection process will begin with a pilot-testing phase conducted to ensure consistent interpretation of the eligibility criteria among reviewers and refine the screening criteria prior to formal screening. During this phase, a random sample of 20 citations from the search results will be independently screened by 2 reviewers based on titles and abstracts. The interrater agreement will be calculated using the Cohen κ statistic.

A κ value of 0.75 or higher will be considered indicative of substantial agreement and sufficient for proceeding to formal screening. If the κ value is below 0.75, the reviewers will discuss the discrepancies, receive additional training on the eligibility criteria, and refine the screening guide if necessary. This pilot process will be repeated with a new sample until the satisfactory agreement level is achieved.

Following the successful pilot test, the study selection process will be performed independently by 2 reviewers. They will first screen the titles and abstracts of all retrieved records against the eligibility criteria using the Rayyan (Qatar Computing Research Institute) or EndNote (Clarivate Analytics) software. The full texts of potentially relevant studies will then be retrieved and assessed for final inclusion. Any discrepancies at any stage will be resolved through discussion or, if necessary, adjudication by a third senior reviewer.

#### Data Extraction

Data extraction will be performed using a standardized, prepiloted data extraction form that will be developed in Microsoft Excel. Two reviewers will independently extract data from the included studies, with any discrepancies resolved through consensus or consultation with a third reviewer. The extracted information will encompass study characteristics such as the first author, publication year, country, sample size, and study design, as well as participant details (age, gender, diagnostic criteria, baseline severity, and comorbidities).

Regarding intervention and comparator details, the reviewers will record the type of massage or *tuina*, specific techniques, acupoints, treatment duration, session frequency, total sessions, and cointerventions (including the systematic recording of the type, frequency, and intensity of all concurrent interventions reported in each study).

Outcome data extraction will involve collecting means, SDs, or change scores from baseline for all primary and secondary outcomes at all reported time points, as well as the number of events for dichotomous outcomes. Furthermore, the specific cognitive assessment tool or specialized scale used for each relevant outcome in each study will be systematically recorded to ensure a nuanced evaluation of cognitive domains.

Finally, safety data concerning the type and incidence of adverse events will be documented, and in instances in which critical data are missing or unclear, the corresponding authors will be contacted via email twice over a 2-week period to request the information.

### Risk-of-Bias Assessment

The risk of bias for each included study will be independently assessed by 2 reviewers using version 2 of the Cochrane risk-of-bias tool for randomized trials (RoB 2) [33]. Any disagreements will be resolved through discussion or consulting a third reviewer. The overall risk of bias for each study and for each domain across studies will be rated as “low risk,” “some concerns,” or “high risk.” The assessment will cover 5 domains.

The first domain is bias arising from the randomization process. We will evaluate whether the allocation sequence was random, whether it was concealed until participants were enrolled, and whether any baseline imbalances suggest a problem with the randomization process. A study will be rated as having “some concerns” if important baseline imbalances in age, gender, or ASD severity are present but not adjusted for in the analysis.

The second is bias due to deviations from the intended interventions. This domain assesses the effect of assignment to the intervention, including whether participants, personnel, and trial contexts were sufficiently blinded.

The third is bias due to missing outcome data. We will evaluate whether the data for the cognitive outcomes (eg, ABC and CARS scores) were complete and whether any missing data could have biased the results.

The fourth is bias in measurement of the outcome. This domain focuses on whether the assessment of cognitive domains (eg, memory and attention) was influenced by knowledge of the intervention, particularly by the outcome assessors.

The fifth is bias in the selection of the reported results. We will examine whether the reported results were selected based on the findings from multiple outcome measurements or different analyses, assessing for potential selective reporting.

Separate from the RoB 2 tool, we will systematically examine the source of funding and the authors’ declarations of interest. Studies funded by organizations with potential commercial or ideological interests in the results, such as massage device manufacturers or specific therapy associations, will be highlighted. A study will be categorized as having significant concerns if there is evidence that the funder influenced the study design, analysis, or reporting.

### Data Synthesis and Analysis

#### Software

Statistical analyses will be performed using RevMan (version 5.4; The Cochrane Collaboration) and Stata (version 17; StataCorp).

#### Meta-Analysis

For continuous outcomes (eg, ABC and CARS scores), the mean difference or standardized mean difference with 95% CIs will be calculated [33]. We prefer using change-from-baseline scores (with SDs of the change) for continuous outcomes; if unavailable, posttreatment scores will be used, and a sensitivity analysis will be performed to assess the impact of this choice.

For dichotomous outcomes (eg, efficacy rates), the odds ratio with 95% CIs will be used.

The decision to perform a meta-analysis will depend on both clinical and statistical heterogeneity. We will first evaluate the clinical similarity of the included studies. A quantitative synthesis (meta-analysis) will only be conducted if the clinical heterogeneity is deemed acceptable. If the interventions are considered too diverse (eg, vastly different techniques or contexts) or if the *I*^2^ value remains high despite subgroup investigations, a descriptive qualitative synthesis will be provided instead of a pooled effect size. The fixed-effects model will be considered only if there is minimal statistical heterogeneity.

#### Subgroup Analyses

If substantial heterogeneity (*I*^2^≥50%) is detected, we will perform subgroup analyses based on several prespecified factors to explore the potential sources of variability. These factors will include participant age tiers, specifically early childhood (<6 years), school age (6-11 years), and adolescence or adulthood (≥12 years) [34]. Additionally, we will examine the type of massage therapy by comparing TCM-based vs Western-based manual therapies and categorize outcomes by specific treatment durations. Outcomes will also be analyzed separately according to time point intervals, specifically immediately after treatment, short-term follow-up (≤3 months after treatment), and medium- or long-term follow-up (>3 months after treatment). Finally, the analyses will account for the comparator type, such as usual care vs placebo, and the intervention context, identifying whether massage therapy was administered as a stand-alone treatment or as an add-on therapy to usual care.

#### Meta-Regression

If significant heterogeneity (*I*^2^≥50%) persists after the aforementioned subgroup analyses, we will conduct univariate random-effects meta-regression to quantitatively explore the sources of heterogeneity. The following categorical variables, derived from the subgroup analyses, will be tested as potential moderators: patient age, categorized as less than 6 years vs 6 years and above; the total treatment course, categorized as less than 8 weeks vs 8 weeks and above; and the type of control intervention, such as usual care vs placebo or sham therapy. Additionally, the proportion of participants receiving a specific concurrent therapy will be evaluated as a moderator to further investigate the robustness of the findings.

#### Heterogeneity

Statistical heterogeneity will be assessed using the *I*^2^ statistic and the Cochran *Q* test (with a significance level of *P*<.10). Given the inherent clinical diversity in massage techniques (eg, TCM based vs Western based), treatment durations, and participant characteristics across the included trials, a random-effects model will be applied as the primary analytical approach for all outcomes. This model is preferred as it accounts for both within-study and between-study variability, providing more conservative and generalizable estimates. The fixed-effects model will only be used as a sensitivity analysis to test the robustness of the results when statistical heterogeneity is absent (*I*^2^=0%) and the studies are deemed sufficiently similar in their population, intervention, comparator, and outcome elements.

Subgroup, sensitivity, and meta-regression analyses will be performed to explore heterogeneity. Following Cochrane recommendations, subgroup analyses and meta-regression will only be performed if at least 10 studies are available for the specific outcome to avoid spurious findings. Results from analyses with fewer than 10 studies will be interpreted with extreme caution, focusing primarily on descriptive presentation.

#### Sensitivity Analyses

To test the robustness of the primary meta-analysis results, sensitivity analyses will be conducted by sequentially excluding studies based on specific criteria and comparing the pooled effect sizes. Specifically, we will exclude studies judged to have a high risk of overall bias, those with a single-group sample size of fewer than 20 participants, and those with a follow-up loss rate greater than 15%. Additionally, we will examine the robustness of the overall findings by including only studies that use the most frequently reported massage modality, such as *tuina* only or Swedish massage only, depending on the identified evidence base. Finally, studies involving adult populations (aged ≥18 years) will be excluded to assess whether the findings remain robust specifically for the pediatric and adolescent ASD population.

#### Publication Bias

If 10 or more studies are included in a meta-analysis, publication bias will be assessed visually using funnel plots and statistically using the Egger linear regression test.

If funnel plot asymmetry is suggested and the Egger test indicates statistical significance (*P*<.05), the nonparametric trim-and-fill method will be used to estimate the number of potentially missing studies and compute an adjusted effect size. The original and adjusted effect estimates will be compared to evaluate the potential impact of publication bias on the results.

### Certainty of Evidence

The certainty of the evidence for the primary outcomes—cognitive and language domain scores from the ABC, CARS, and ATEC scales—will be assessed using the Grading of Recommendations Assessment, Development and Evaluation (GRADE) approach. The evidence will start as “high certainty” for randomized trials and may be rated down by one or more levels based on the following 5 domains.

First, regarding the risk of bias, the certainty will be downgraded if 50% or more of the participants contributing to the outcome are from studies judged to be at a high risk of bias according to the RoB 2 tool.

Second, indirectness will be considered, with the certainty rated down if significant concerns arise regarding the population, intervention, or outcomes. Specifically, the certainty will be rated down for indirectness of the population if most (>50%) of the included evidence is derived from adult ASD populations despite the target population of the review encompassing all ages.

Third, the presence of substantial unexplained heterogeneity, such as an *I*^2^ value of 50% or more, a low *P* value from the chi-square test, or nonoverlapping CIs, will lead to a downgrade for inconsistency.

Fourth, imprecision will be evaluated, where a downgrade will occur if the total sample size is smaller than the optimal information size (typically considered as <400 participants for continuous outcomes) or if the 95% CI around the pooled effect estimate includes both a potentially important benefit and no effect (the null value of zero for mean differences).

Fifth, the certainty will be downgraded if there is significant evidence of publication bias indicated via funnel plot inspection and a *P* value of .05 or lower in the Egger test.

Two reviewers will independently assess the certainty for each key outcome. The final assessments (high, moderate, low, or very low) will be presented in a “summary of findings” table.

### Ethical Considerations

As this study is based exclusively on analysis of previously published literature, ethics approval is not required. The final results will be submitted for publication in a peer-reviewed journal and presented at relevant academic conferences. Any modifications to the study protocol will be documented and updated in the PROSPERO registry.

## Results

### Study Selection

**Figure 1.**
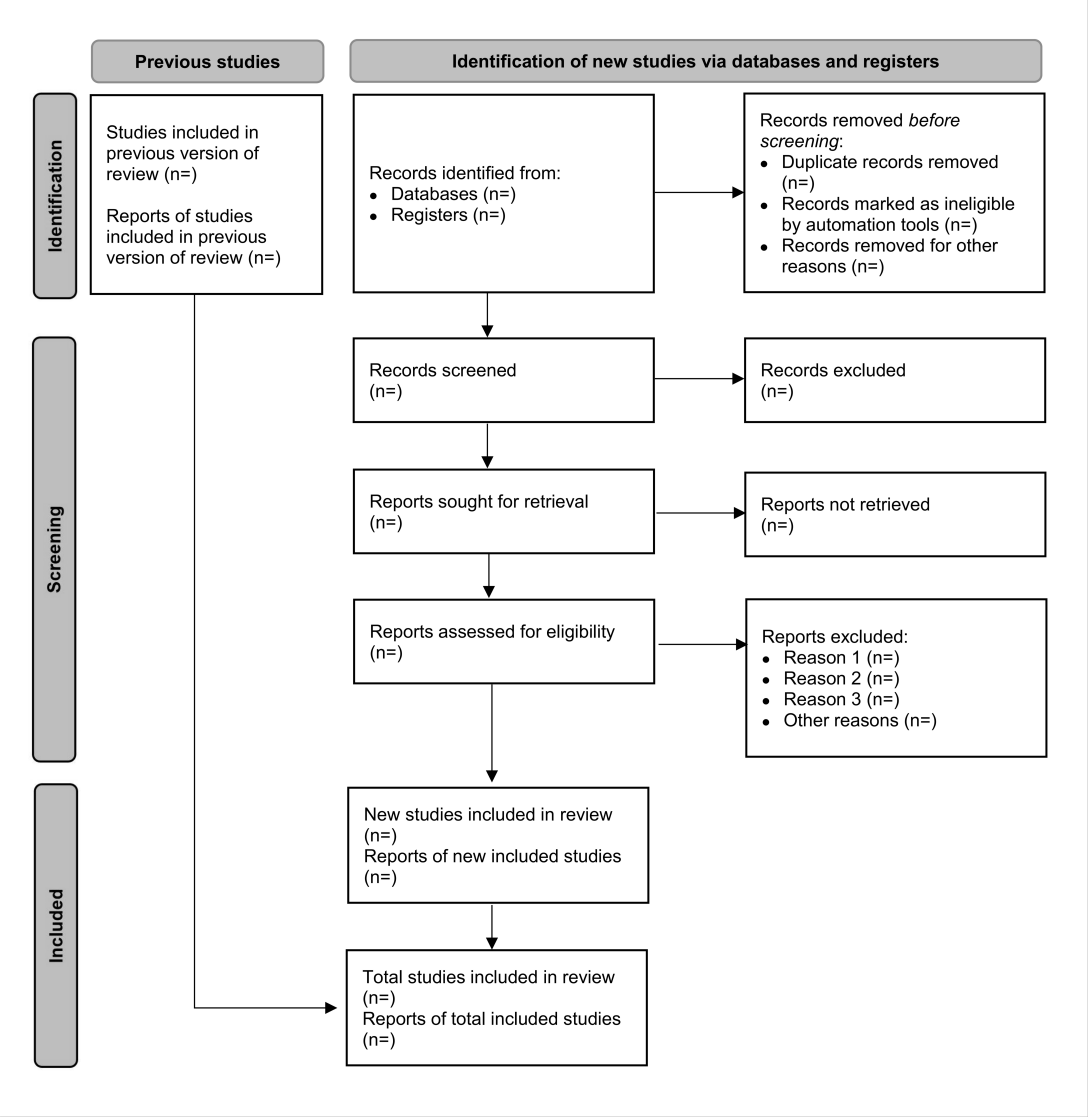
PRISMA (Preferred Reporting Items for Systematic Reviews and Meta-Analyses) flow diagram of the study selection process.

The results of the literature search and the process of study selection (including screening, eligibility assessment, and final inclusion) will be documented and presented in a PRISMA (Preferred Reporting Items for Systematic Reviews and Meta-Analyses) flow diagram, as shown in Figure 1.

### Current Status

This study was funded in 2024. The literature search was initiated in early 2025 and concluded in September 2025. As of September 2025, the comprehensive literature search across eight electronic databases yielded a total of 994 records. Among the English-language resources, the search identified 40 results from PubMed, 104 from Web of Science, 149 from Scopus, and 240 from the Cochrane Library. Regarding the Chinese-language databases, 113 records were retrieved from the CNKI, 159 from Wanfang Data, 97 from the VIP Chinese Scientific Journal Database, and 92 from the Chinese Biomedical Database. These results provide the foundation for the subsequent study selection process as illustrated in the PRISMA flow diagram. Study selection, data extraction, and analysis will be completed by June 2026. The final systematic review is expected to be submitted for publication in December 2026.

## Discussion

### Expected Findings

This systematic review is expected to provide a comprehensive synthesis of the current evidence regarding massage therapy for cognitive impairment in individuals with ASD. We hypothesize that massage therapy, whether applied as a stand-alone treatment or as an adjunct to conventional care, will demonstrate a positive effect on cognitive and language outcomes as measured using standardized scales such as the ABC, CARS, and ATEC [35,36]. Furthermore, we anticipate that the safety profile will be favorable, with few and minor adverse events reported. By targeting specific cognitive domains, this study aims to fill a critical gap in the existing literature regarding nonpharmacological cognitive interventions for ASD.

Previous meta-analyses, such as the study by Fadlalmola et al [30], have focused on behavioral symptoms and parenting stress. However, cognitive impairments—which are a major cause of disability and long-term functional challenges in ASD—remain underinvestigated in the context of manual therapies. This review is distinguished by its exclusive focus on cognitive impairment as the primary outcome, aiming to provide the first systematic synthesis of evidence regarding its effects on specific cognitive functions such as memory and executive function.

A major strength of this study is that it will be the first systematic review specifically targeting cognitive outcomes in ASD. The protocol adheres to the PRISMA-P guidelines and includes a rigorous plan for investigating heterogeneity through subgroup analysis and meta-regression. Potential heterogeneity may arise from the diversity of massage techniques and varying degrees of ASD severity [37]. Additionally, the reliance on published data may introduce publication bias, and the availability of long-term follow-up data may be limited.

The findings of this review will identify specific gaps in the current literature, such as the need for more large-scale, high-quality RCTs with standardized protocols. If efficacy is demonstrated, it may encourage the integration of massage therapy into standardized rehabilitation protocols and support clinical decision-making for health care providers and families seeking complementary therapies. Future research should also explore the underlying neurobiological mechanisms through which tactile stimulation influences cognitive development in ASD.

The final results of this systematic review and meta-analysis will be submitted for publication in a high-impact, peer-reviewed journal specializing in pediatrics, integrative medicine, or neurodevelopmental disorders. Additionally, the findings will be presented at relevant international and domestic academic conferences to reach a broad audience of practitioners and researchers.

### Strengths and Limitations of This Study

This will be the first systematic review and meta-analysis specifically targeting the efficacy of massage therapy on cognitive impairment in ASD, addressing a clearly defined evidence gap by focusing on specific cognitive domains such as executive function, attention, and memory. The analytical framework is strengthened by a predefined and detailed plan for investigating heterogeneity through subgroup analysis and meta-regression, alongside testing robustness via sensitivity analysis. Despite these strengths, potential limitations include high heterogeneity resulting from varied massage techniques and ASD severity; the risk of publication bias, such as the underreporting of negative findings; and the likely scarcity of long-term data on cognitive outcomes.

## Supplementary material

10.2196/88284 Checklist 1 PRISMA-P checklist.

## Abbreviations

ABC: Autism Behavior Checklist
ASD: autism spectrum disorder
ATEC: Autism Treatment Evaluation Checklist
CARS: Childhood Autism Rating Scale
CNKI: China National Knowledge Infrastructure
GRADE: Grading of Recommendations Assessment, Development and Evaluation
PRISMA: Preferred Reporting Items for Systematic Reviews and Meta-Analyses
PRISMA-P: Preferred Reporting Items for Systematic Reviews and Meta-Analyses Protocols
RCT: randomized controlled trial
RoB 2: version 2 of the Cochrane risk-of-bias tool for randomized trials
TCM: traditional Chinese medicine
